# Balancing Selection and Its Effects on Sequences in Nearby Genome Regions

**DOI:** 10.1371/journal.pgen.0020064

**Published:** 2006-04-28

**Authors:** Deborah Charlesworth

## Abstract

Our understanding of balancing selection is currently becoming greatly clarified by new sequence data being gathered from genes in which polymorphisms are known to be maintained by selection. The data can be interpreted in conjunction with results from population genetics models that include recombination between selected sites and nearby neutral marker variants. This understanding is making possible tests for balancing selection using molecular evolutionary approaches. Such tests do not necessarily require knowledge of the functional types of the different alleles at a locus, but such information, as well as information about the geographic distribution of alleles and markers near the genes, can potentially help towards understanding what form of balancing selection is acting, and how long alleles have been maintained.

## Introduction

The concept of balancing selection is well-established, appearing in every genetics and evolution textbook. Classic examples are known in humans and other organisms, and two different forms of balancing selection are very familiar—heterozygote advantage at a locus (often called overdominance), and frequency-dependent selection with a rare-allele advantage (although overdominance is often incorrectly used as synonymous with balancing selection). It is well-known that balancing selection maintains different alleles at the selected loci. A familiar example of frequency dependence (though not always viewed in this way) is the selection on sex ratio that maintains males and females in a population with an X/Y sex chromosome system, which behaves like a single gene, since large parts of the sex chromosomes, including the regions containing the sex-determining region, do not undergo genetic crossing-over.

More complex models of selection maintaining diversity include temporally or spatially heterogeneous selection, which can sometimes maintain different alleles [1,2], and systems with interactions between species (or other genetically interacting systems). An example is male sterility in plants. Maternally transmitted selfish cytoplasmic male-sterility (CMS) factors that increase seed production can invade hermaphrodite populations, but generally cannot spread throughout a population, because, as females become common, hermaphrodites become the only source of pollen to fertilise females' seeds; relative fitness of non male-sterile individuals then increases. Thus, as with the male/female sex polymorphism, a balanced polymorphism is established, with females and hermaphrodites in the population. Restorer alleles, suppressing the sterility of plants with the sterility cytoplasmic genotype, may then often spread. Although fixation is possible, polymorphism may again sometimes remain within populations for both cytoplasmic and restorer genes, with complex frequency dependence leading to stable equilibrium frequencies of the genetic factors, or to stable or slowly decaying oscillations in the frequencies [3].

Host–pathogen systems can behave similarly, with a pathogen invading a host population, thus creating selection for resistant genotypes. Mutant resistance alleles can either become fixed (with, potentially, a succession of such fixations as new pathogens appear, i.e., an “evolutionary arms race”) or establish polymorphisms, with different resistance alleles present within populations or in different populations of a species [4,5].

The timescales of the different kinds of balancing selection determine how the selection affects sequences in nearby genome regions (Table 1). Sex chromosomes are maintained for long evolutionary times, while situations involving pathogens might often be ephemeral, since hosts might evolve resistance alleles that become fixed in the species. Many of the classic cases have now been restudied using DNA sequence data, and clear “footprints” of selection are sometimes found, which can be used to detect selection and to distinguish balancing from other forms of selection. These tests depend on the interaction of balancing selection at amino acids or other sites in genes with recombination, affecting non-selected (neutral) variation in nearby genome regions. This review does not deal with technical aspects of testing for balancing selection, but focuses on an understanding of how such selection affects diversity at neutral sites near sites under balancing selection, and how balancing selection over different timescales leads to different footprints.

**Table 1:**
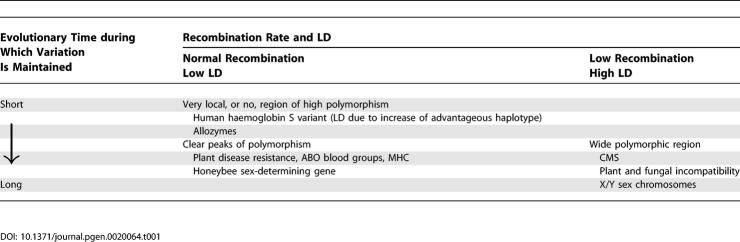
Schematic Classification of Balancing Selection, with Some Examples That Are Discussed in the Text

## Long-Term Balancing Selection: High Sequence Diversity in Genes where Polymorphisms Are Maintained for Long Evolutionary Times

The familiar textbook view of balancing selection stresses the most dramatic cases, with alleles maintained for very long evolutionary times. Balancing selection is often portrayed as “diversifying,” meaning that there is an advantage to new alleles, as with plant self-incompatibility (*S*) alleles, where the frequency-dependent selective advantage of rare pollen and pistil types is well understood to maintain many alleles [6,7], or fungal incompatibility alleles [8,9], whose selective maintenance remains unclear, despite evident similarity to plant SI.

When the same alleles persist for long times, balancing selection may be detectable from its effects at nearby neutral sites. The population genetics of balancing selection shows that, as well as maintaining diversity at the selected sites themselves (generally maintaining different amino acids), it increases diversity at closely linked neutral sites [10–12]. Regions of genome close to a site under balancing selection, which rarely recombine with the selected site(s), will have common ancestors longer ago than other regions (longer coalescence times), because migration of variants between allelic classes depends on recombination. This high diversity is not due to diversifying selection, since systems with just two states, such as sex-determining genes, where selection on sex ratio gives the rarer sex an advantage, with no diversifying selection, can also be maintained in the long term (though sometimes a sex-determination system is replaced by a new one [13]). This example clearly illustrates the evolution of high diversity. The divergence of the X and Y chromosomes were once homologues. With the acquisition of sex-determining functions and loss of recombination, genes on these chromosomes now have, in several taxa, higher sequence divergence than between related species [14–16].

If different functional types of alleles at a locus persist long enough, each allele class can acquire its own unique set of neutral mutations, each associated with the class in which it arose, until eventually recombination causes “migration” into a different allele (reviewed in [17]). The region around alleles of functionally different types can thus differ at multiple non-selected sites, so that polymorphism will be higher than in unlinked genome regions, over a distance depending on the local recombination frequency, and variants in the region will show linkage disequilibrium (LD) due to associations between functionally different alleles [11,18].

High diversity can thus provide evidence for balancing selection. In plant species with CMS, large frequency differences of females in natural populations, and differences in the frequency of restoration of male fertility when females from one population are pollinated by males from elsewhere, indicate highly variable frequencies of the genetic factors involved. This might reflect regular turnover of the sterility and restorer factors, in an arms race [19], or perhaps frequency oscillations [3,20]. However, high diversity has been found in sequences of a mitochondrial gene within populations of *Silene acaulis,* a plant with CMS [21], which excludes turnover of cytoplasmic genotypes, or in prolonged periods of low frequency for any of these genotypes. In this species at least, the male-sterility polymorphisms must therefore have been maintained for long times.

**Figure 1:**
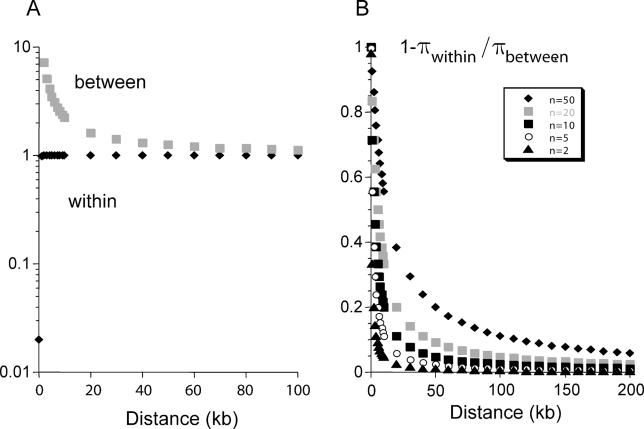
Sequence Diversity Expected at Neutral Sites at Different Distances from a Site under Balancing Selection The figure shows the dependence of diversity at neutral sites in a gene on the number of different alleles maintained (*n* values) and the distance from the selected sites. A recombination rate of 1 cM/Mb is assumed, which is appropriate for humans, but much lower than the estimated rate for A. thaliana or maize. The example calculated is based on equations in the Appendix of [12], which are appropriate for selection at loci, such as MHC, where homozygous genotypes can be formed (e.g., a system with heterozygous advantage in which homozygotes are viable); note, however, that heterozygous advantage is unlikely to maintain very large allele numbers [35,82]. In the example shown, the turnover rate of alleles at the selected locus (or site) is assumed to be 10^−7^. (A) Shows predicted nucleotide diversity (π) between and within haplotypes of allelic classes (defined as having different alleles at the selected site or sites) for the case when 50 different alleles are maintained. (B) Shows the proportion of the overall diversity that is between allelic classes (analogous to F_ST_ in a subdivided population), showing differentiation between the haplotypes across several kb when there are many alleles, even when recombination occurs.

The CMS case is extreme, because, like sex chromosomes, mitochondrial genomes rarely recombine, since heteroplasmy is rare. Even with recombination, however, considerable sequence diversity can exist several kilobases from a selected site, in systems with many different alleles (Figure 1). Long-term maintenance of honeybee sex-determining alleles may be one such case, with high amino acid and synonymous site diversity [22]. Nucleotide diversity is also extremely high throughout the sequences of multi-allelic pistil recognition genes of plants with gametophytic self-incompatibility, e.g. [23–25], and in the pistil and pollen *S*-loci of species with sporophytic incompatibility [26,27]. Recombination rates between the pollen and pistil *S*-loci are not known, but may be low, because selection against self-fertile recombinants is likely to be strong.

If host–pathogen co-evolution leads to long-term maintenance of variation, this should therefore be detectable from these “footprints” at nearby silent sites and marker loci, even if we are unable to classify the functional types of alleles and determine their number (though fewer alleles are expected than for incompatibility loci). Some loci known to be involved in defence processes indeed have high sequence polymorphism. One such locus in *Arabidopsis thaliana,* is estimated to have nucleotide diversity above 4% for synonymous sites, and even for non-synonymous ones [28], much above the average for this species (<1% for synonymous sites [29]). These genes are difficult to study, because they are often members of gene families, and it is essential in studying polymorphism to be sure that the sequences are from a single locus, and to exclude “migration” from paralogous genes, which might occur by gene conversion or other exchange processes.

If exchanges between alleles are frequent, or allele numbers are not large, even long-term balancing selection causes high differentiation between alleles only very close to the selected sites [12,30], while exchanges erode differences at synonymous and intron sites elsewhere in the gene (Figure 1). It may thus be difficult to distinguish between long-term balancing selection with recombination, and short-term maintenance of alleles (the likely situation for allozyme loci, discussed later). Recombination also implies that tests may fail to detect selected loci by searching for high diversity genes, e.g., [31]. Loci will be missed where selection has not acted for long enough, or exchanges are too frequent, to allow for diversity to build up between alleles.

Recombination clearly occurs at the histocompatibility (MHC) loci [32–34]. Although their diversity per nucleotide site is only a few percent [33], this is exceptionally high for human sequences (though much lower than diversity in plant or fungal incompatibility gene sequences). These genes' much-cited high allelic diversity largely results from recombination between differentiated haplotypes, and this differentiation clearly indicates long-term balancing selection. Arguments against MHC alleles being maintained by overdominance are based on the difficulty of maintaining large allele numbers [35], but although numbers of functionally different alleles are currently unknown they must be lower than haplotype numbers.

## Trans-Specific Polymorphism

**Figure 2:**
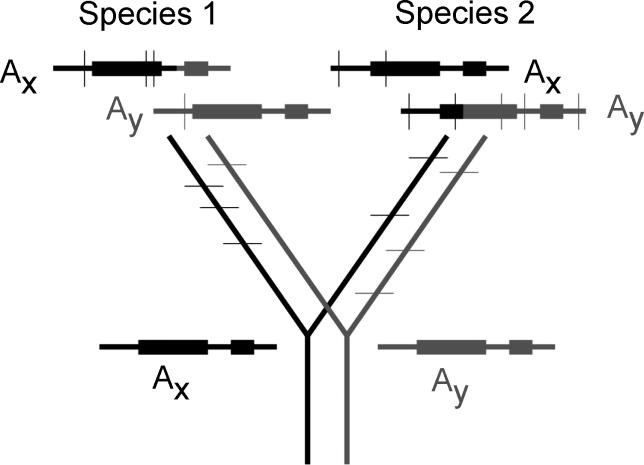
Lineages at a Locus under Long-Term Balancing Selection Two haplotypes with different alleles, A_x_ and A_y_, which diverged before the common ancestor of two species (1 and 2), are denoted by black and grey lines and boxes, respectively (denoting genes). Variants in the regions in and around the selected locus will remain associated with the haplotype in which they arose until recombination occurs with a different haplotype, even after the species become isolated. Species–specific differences (shown as thin horizontal lines in the tree and vertical lines in the haplotypes) will also accumulate. Recombination between different haplotypes (indicated by mixed black–grey haplotypes) will mean that sites close to the selected sites will be most differentiated between alleles (see Figure 1).

Another effect of long-term balancing selection, also relying on the evolution of highly differentiated alleles, is trans-specific polymorphism. When the same alleles persist for long times, and are not regularly replaced by new alleles (“turnover”), allele ages can exceed the ages of related species [36]. If a species with such a balanced polymorphism splits into two, multiple different haplotypes will often pass to the daughter species. Figure 2 gives a hypothetical example for sites in and near a gene. Initially, the associations of variants will be the same as in the ancestor, but, over evolutionary time, this signal will become indistinct, as the sequences of each daughter species' copies of each allele type recombine with other haplotypes of the locus, acquire new mutations, or are lost or evolve into new, functionally different alleles (allele turnover). After enough time, the sequences will cluster by species, rather than by functional types.

Trans-specific polymorphism is highly unlikely under neutrality, except between species that are so closely related that they are likely to share variants present in their common ancestors, so it should provide a test for long-term balancing selection [18]. For plant and fungal incompatibility systems, the same types are sometimes detectable in different species [8,9]. In Brassica oleracea and *Brassica rapa, S*-alleles with similar sequences of the pistil receptor gene reject each others' pollen [37]. Even in incompatibility systems, however, typing is laborious, and most analyses infer trans-specific polymorphism from gene trees using sequences from multiple species (e.g., [23,38–40]). When sequences do not cluster by species, long-term maintenance of alleles is likely. However, reconstructing trees is inappropriate, since sequences may recombine within the study species or their ancestors [41], and, in the absence of functional information, trans-specific or trans-generic alleles are determined arbitrarily. The signal of fixed differences between species also quickly overwhelms that of shared variants unless sequences of functionally different alleles differ hugely, as in MHC systems [42]. Ideally, individual variants in sequences should be examined to test rigorously for unexpected numbers of trans-specific polymorphisms [43].

In most cases, we do not know the nature of the selection maintaining polymorphisms with multiple alleles, except in very general terms (e.g., MHC polymorphisms may be connected with resistance to diseases), and therefore cannot generally classify alleles into functional “types” and recognise when the same functional alleles are shared between different species. It is extremely complex to determine the effects of amino acid changes in the peptide binding regions of MHC proteins on the strength and specificity of their binding to peptides. However, shared amino acid sequence motifs determining similar binding properties between primate species are sometimes recognisable [44]. Even this does not necessarily imply long-term balancing selection. Despite apparently similar ABO blood groups in different primate species and high sequence diversity [45], the sequences of A, B, and O alleles have few trans-specific variants, so recombination may occur between alleles, and convergent evolution between species has been suggested [46].

Tests for trans-specific polymorphism at silent sites in sequences should nevertheless help us to detect long-term balancing selection, even without being able to classify alleles functionally. Searching human and chimpanzee gene sequences for trans-specific polymorphism, we uncovered little evidence for long-term balancing selection, except for MHC sequences [43,47]. For MHC genes, frequently observed high diversity [48] and trans-specific polymorphism [42] rule out a high turnover rate and, thus, arms-race scenarios, though this does not necessarily suggest overdominant selection.

## Short-Term Balancing Selection

Long-term balancing selection is, however, probably unusual. The evolutionary lifespans of alleles (or, inversely, their turnover rates) are likely to be very important in understanding pathogen systems, in which frequency-dependent selection can sometimes maintain allelic diversity, but directional selection for resistance involving arms races may sometimes occur. Estimating diversity at known disease-resistance loci, without knowing the alleles' functional types, or even the relevant pathogens in nature, suggests that some of them maintain long-term polymorphisms [49,50].

Even with directional selection due to pathogens (or to human disturbance, e.g., a pesticide), polymorphisms may establish, because heterozygote advantage can arise simply from a disadvantage of a new allele when homozygous. When a resistance mutation arises, if heterozygotes are resistant, and have no other strong disadvantage, the allele will increase in frequency. In an outcrossing population, homozygotes for the mutation are initially rare. Consequently, even a strong survival or fertility disadvantage of the mutation in homozygotes cannot prevent its increase to an intermediate frequency, but may lead to a balanced polymorphism.

**Figure 3:**
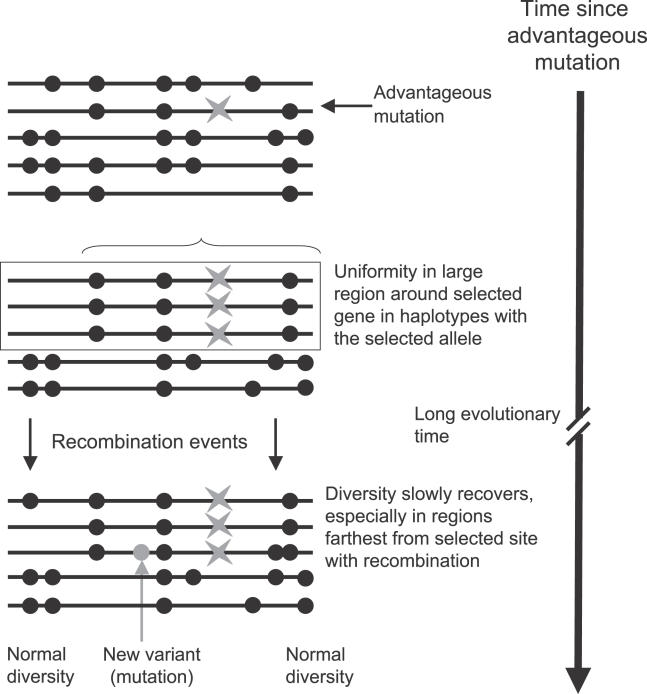
Haplotypes in a Genome Region after Spread of an Advantageous Mutation That Establishes a Balanced Polymorphism An advantageous mutation (denoted by the star) arises and quickly spreads to a high frequency. Variants (black dots) in the region of genome around the selected site will be carried to high frequency in the haplotypes with the mutation, and recombination subsequently introduces variants from the rest of the population, especially at sites distant from the selected site. Mutations may also occur. Note that the hitchhiking does not contribute to differentiation between haplotypes, since the variants were present before the selective event.

When such alleles arose recently, the sequences at the locus can show a characteristic pattern in which the new alleles are uniform throughout a large region surrounding the gene. A mutation with a strong selective advantage, which increases in frequency rapidly, has too little time to recombine with variants in the surrounding region of genome, or to incorporate variants by mutation, especially in the case of partially dominant advantageous alleles, which quickly increase in frequency [51]. Low diversity due to such “selective sweeps” (Figure 3) is the basis of one type of test for recent spread of advantageous allele, using silent site variants in the sequence of the locus itself and its introns, e.g., [52–54], or markers such as microsatellite alleles at closely linked loci [55].

When selection opposing fixation has led to a recently established balanced situation, the initial sweep (or hitchhiking) event is potentially detectable from “homozygosity” of variants in and around the locus, since it creates a high frequency of one haplotype [53,56]. Such footprints of recently increased frequency of a uniform haplotype are evident near the β-globin locus in African populations with the classic balancing selection example, sickle-cell allele [57], and across large regions of the chromosome carrying the rat warfarin-resistance gene [58]. The regions affected by such selective sweeps are generally much larger than the region of LD around a locus under long-term balancing selection, because recombination has not yet eroded differences between the selected allele and others, but when the advantageous allele is maintained by balancing selection and does not become fixed in the population, diversity is severely reduced only in haplotypes carrying that allele (Figure 3). Other previously known cases of balancing selection showing such patterns include the human polymorphisms PTC taster/non-taster [59], glucose-6-phosphate dehydrogenase alleles [60], and haemoglobin E, another globin variant involved in resistance to malaria [61]. Allozyme polymorphisms may also be due to recent selective events, and the classic case of balancing selection, *Drosophila* inversion polymorphisms [62], may also often not be maintained for very long times [see 63–65].

It might seem to be straightforward to discover new examples of selection by using these signs of selection in genome scans. However, rapidly increased frequency of one allele at a locus occasionally happens by chance, as genetic drift occurs in a population, and will be indistinguishable from selection events. Thus, tests must also show that loci identified lie outside the bounds of what might occur if the variants were neutral. This is difficult, because real populations are often subdivided, and the sub-populations (and thus the species as a whole) have unknown complicated histories involving size changes and migration, which cannot be taken into account [66]. This is proving to be a problem for inferring selection in human populations, despite very large studies [67,68], and it surely applies to other populations. It may be helpful to compare the extent of uniform haplotypes of different alleles [53] or many different loci [54], which should often share similar histories [69]. Certainly, given the problems for established tests for selection including McDonald–Kreitman, Hudson, Kreitman–Aguadé, and Tajima's tests [reviewed in 70] that can be caused by unknown population subdivision and history [71–73], these tests are now often supplemented by other evidence.

The difficulties are greatest for weak selection, or selection events that occurred long ago. Weak balancing selection may often occur, and could be the basis of much quantitative variability, including variation in fitness. In finite populations, fixation can occur for alleles under weak balancing selection, which can complicate tests for selection [74].

## Local Adaptation

There is much evidence from whole organism studies, such as reciprocal transplant experiments, for selective differences between populations [75], which could create balancing selection at the metapopulation scale. The detailed genetic basis of such local adaptations is interesting, as is the duration of differences. Estimates of genetic subdivision may be helpful, particularly F_ST_, which estimates the proportion of diversity between populations. F_ST_-based tests for selection are already in use [76,77]. This approach should help with an understanding of selection of host–pathogen systems in natural populations, which may often involve local adaptation [e.g., 78]. If sequences of loci involved in pathogen defences suggest unusually high subdivision compared with other loci, this might suggest selection for locally adaptive alleles, differing from one population to another. In contrast, loci where balancing selection maintains alleles within populations, such as CMS haplotypes, or incompatibility loci, should show less evidence for subdivision than the average locus [73,79]. This approach may be helpful in understanding selection on MHC genes [80]. Due to recombination, different populations will generally have different sets of alleles, but sequences should reveal whether populations share variants or differ significantly more than other loci (suggesting local selection differences). In an MHC locus studied in deer mouse populations, low F_ST_ was found, suggesting similar selection across populations [81].

## Conclusions

Analyses of DNA sequences have the promise to advance understanding of the different forms of balancing selection. Sequences can uncover highly polymorphic loci (even in the presence of recombination), define regions of LD and detect trans-specific polymorphism of neutral variants. It will be particularly interesting to combine such results with F_ST_ estimates from sampling multiple natural populations, to test which cases involve maintenance of diversity within populations, and which do not. Even if they include false positives, sequence-based tests can provide interesting sets of genes that may be under selection, of interest for detailed studies. 

## Abbreviations

CMS: cytoplasmic male sterility
LD: linkage disequilibrium
MHC: histocompatibility
